# Effects of the War on National Insurance

**Published:** 1914-12-19

**Authors:** 


### NATIONAL INSURANCE ACT DEVELOPMENTS.

### Effects of the War on National Insurance.

The war has affected the operation of the National Insurance Acts in more ways than are generally realised. The effects have been both direct and indirect, and it will undoubtedly be many years before the full results will be manifest in all their bearings. It will be useful, therefore, if we endeavour to give some idea of the effects of the war on National Insurance in so far as they can at present be appreciated. Among the direct results of the war the first place is taken by the National Insurance (Navy and Army) Act, 1914. We have already referred to this measure in our columns, but at that time only the text of the Bill had been published, and the important regulations which have since been issued by the Insurance Commissioners in reference to the matter call for brief comment.

### National Insurance (Navy and Army) Act.

It will be remembered that the Act provided that soldiers specially enlisting for the war and all previously insured persons who serve during the war as commissioned or warrant officers of the Naval Reserves, or officers of the Reserve or of the Territorial Force, or are granted temporary commissions in the Regular Forces during the war are to come within the operation of Section 46 of the Act of 1911, as that section applies to men of the Territorial Forces called out on embodiment. The direct result of this enactment is that the men in question are to be treated in regard to National Insurance in the same way as soldiers—that is to say, the only benefit to which they are immediately entitled is maternity benefit, while, on the other hand, their contributions are reduced to 1½d. a week. Section 46 of the Act of 1911, however, allowed the application of the provision for men of the Territorial Force called out on embodiment to be modified or adapted to meet special circumstances as might be prescribed by the Insurance Commissioners. In the exercise of their right to make such regulations the Insurance Commissioners have prescribed that only those who before joining the Colours were insured are compulsorily insurable while they are so serving. Thus it is made optional to a man who joins the Forces for the war whether he will or will not become insured if he has not already been an insured person in civil life. It will at once be seen that the regulations are designed to protect the insurance interests of those who were already insured, while at the same time they give freedom of choice to those who upon return to civil life may not have the right to continue in insurance.

### The Position of Nurses.

Another direct result of the war is the employment of insured nurses with the Forces, and we pointed out immediately after the outbreak of hostilities that their case called for special treatment, seeing that while they are so serving they are not in need of the benefits provided by their insurance. We now learn that the Insurance Commissioners have decided that no Health Insurance contributions are required to be paid, either by or in respect of nurses to His Majesty's Forces, who are employed by the Army Council for service during the war. A certificate of exception from insurance has been issued, as allowed by the Act of 1911. In order that this action may not operate to the detriment of the insurance interests of the nurses in question after the war, we understand that the Army Council has agreed to pay in respect of these nurses when they leave their Army employment, otherwise than through misconduct, such lump sums as will enable them to resume their insurance without any loss of benefit. The certificate of exception does not apply to nurses employed by the Red Cross Society or other voluntary agencies, but it may be pointed out that while such nurses are abroad no contributions are required to be paid. When they return to normal employment in the United Kingdom it would seem that the disabilities attaching to arrears of contribution will apply unless a scheme is adopted for paying such lump sums as will enable them to resume their insurance without loss of benefit.

### The Relief of Distress Through Unemployment.

Although the present scheme of insurance against unemployment is of much more restricted scope than that of health insurance, it has already been made abundantly clear that the relief of distress through unemployment due to the war has been made easier through the existence of the insurance fund. To assist it further, the Board of Trade have put forward a scheme under which emergency grants will be made to voluntary associations which provide benefits for their unemployed members. This is another direct result of the war, and in it may be descried the possible extension at an early date of the unemployment provisions of the Act to other than the trades to which they at present apply, for it will be noticed that the grants are not restricted to the present insured trades. It is true that a similar scheme has already been available under section 106 of the Act of 1911, whereby any association not trading for profit, and distributing payments to unemployed members, could claim, under certain conditions, the refund of an amount not exceeding one-sixth of that expended on such payments during the preceding year. The emergency grants are, however, much more attractive both as to their amount and as to the conditions under which they can be claimed. The conditions are: (1) That the association should be suffering from abnormal unemployment; (2) that the association should not pay unemployment benefit above a maximum of 17s. a week, including any sum paid by way of State unemployment benefit; and (3) that the association should agree, while in receipt of the emergency grant, to impose levies over and above the ordinary contributions upon those members who remain fully employed.

The emergency grants are either one-sixth or one-third of the amount expended in benefits, in addition to the one-sixth already obtainable under the existing scheme. Thus the total amount of the refund will be either one-third or one-half of the payments made. The choice of scale will depend upon the decision of the association as to the amount of the special levies which it will make on the employed members. When, for instance, the maximum rate of unemployment benefit paid by the association is 15s. a week, the rate of special levy required to obtain an emergency grant of one-sixth is 2d. a week, and for a grant of one-third it is 4d. a week.

It is thus clear that for comparatively small sums to be paid by those who are so fortunate as to retain their employment their associations can obtain for those who are out of employment substantial relief. The cost to the Government will, of course, depend upon the extent to which the scheme is adopted, but it is not thought that the amount will be more than £200,000 or £300,000, and as this sum will be expended by organised associations there can be no doubt that the money will be wisely administered.

### Indirect Effects of the War.

The indirect effects of the war upon national insurance are numerous. Some of them have already come into operation, while others can only be dimly discerned. One of the immediate results has been the increase in the price of drugs, and this has thrown into prominence the question of the remuneration of the chemists from the medical benefit funds. The rise in prices is being used as an additional argument for the revision of the system under which the payments out of the drug fund are at present made.

Another similar effect will be manifest at an early date, because the transfer of so many insured persons from the ordinary class to the Army and Navy class, and the consequent lapse of the right to benefit, will remove the names of these men, at least for the time being, from the lists of their panel doctors, whose remuneration will accordingly be reduced. In this connection it may be pointed out that even though a soldier may remain living at home for some time after his enlistment, as has been the case with some of the more recent levies in certain Territorial units, he is not entitled to the services of his panel doctor, for the right to medical benefit under the National Insurance Act ceases upon enlistment. The same remark applies with regard to the medical treatment of a soldier invalided home. He is not entitled to medical treatment from his panel doctor, and such treatment as may be necessary will be given by the medical officer of his unit. When the man's home is some considerable distance from headquarters, it would be a convenience if some arrangement could be made to secure the services of his panel doctor.

Another indirect effect of the war on national insurance is the absence of many of the officers of approved societies from their usual posts. Officers of societies have enlisted in the newly raised forces, and if any slight inconvenience or delay is occasioned to members in the settlement of claims it may well be that the cause is to be sought in the shortage of officers and staff of the society. In spite of this depletion of the usual staffs of societies, we believe that the work is, on the whole, being carried out very much as usual, though the completion of the returns required by the Insurance Commissioners for the purpose of allocating the initial reserves, and as a basis for the valuation of the societies, must necessarily be somewhat retarded. It must not be overlooked, moreover, that the change of so many insured men from the ordinary insured class to the Navy and Army class will necessarily involve a large amount of clerical labour, with a corresponding amount of trouble when, at the end of the war, the retransfer has to be made. Seeing that the normal amount allowed by the Insurance Commissioners to the approved societies to cover the administrative expenses of the Navy and Army class is much less than the amount allowed for ordinary employed contributors, it would seem that the societies, or some of them, will be called upon to make drastic alterations in their expenditure, or some special allowance will have to be made by the Commissioners to enable the societies to cover the inevitable deficiencies in their administration accounts.

### Is the Financial Soundness of Approved Societies Affected?

This brings us to the last point which we have space to consider, and that is the ultimate effect of the war on the finance of national assurance as at present administered by Approved Societies. At the National Conference of Friendly Societies, held at Norwich in October, the President is reported to have said: "The financial basis of the Insurance Acts is bound to be considerably affected. The younger and more healthy lives are those which are being sent to the Front; many of these must return more or less incapacitated, and with constitutions weakened; therefore they will be claimants upon the fund many years before the actuarial expectation has been reached. The contributions paid will, by reason of unemployment, be considerably reduced, and thus both sides of the account will be prejudicially affected." The facts as here stated are incontestable, but it does not follow at once that the financial soundness of the societies will be impaired. It is possible that the release of the reserves that will be made, in advance of the actuarial expectation, by the deaths of those who fall in action or who succumb to their wounds will counterbalance the prejudicial effects outlined above. Time and the actual tabulation of experience alone can show the ultimate effect of the war in this direction, and it may be many years before the full results can be ascertained. In the meantime there is no reason to suppose that the insurance funds of the societies are in any immediate danger of insolvency, and the suggestion that an outcome of the war will be the necessity for the State to take direct control of the administration of the National Insurance benefits is premature.

